# Delayed hospital admission for traumatic hip fractures during the COVID-19 pandemic

**DOI:** 10.1186/s13018-021-02382-w

**Published:** 2021-04-01

**Authors:** Stephanie Jarvis, Kristin Salottolo, Robert Madayag, Jennifer Pekarek, Nnamdi Nwafo, Alexander Wessel, Therese Duane, Zachary Roberts, Mark Lieser, Chad Corrigan, David Bar-Or

**Affiliations:** 1 ION Research, 501 East Hampden Avenue, Englewood, Colorado 80113 USA; 2 St. Anthony Hospital, Lakewood, CO USA; 3 Swedish Medical Center, Englewood, CO USA; 4 Penrose Hospital, Colorado Springs, CO USA; 5 Medical City Plano, Plano, TX USA; 6 Research Medical Center, Kansas City, MO USA; 7 Wesley Medical Center, Wichita, KS USA

**Keywords:** Hip fracture, Time to arrival, COVID-19

## Abstract

**Background:**

Concerns of contracting the highly contagious disease COVID-19 have led to a reluctance in seeking medical attention, which may contribute to delayed hospital arrival among traumatic patients. The study objective was to describe differences in time from injury to arrival for patients with traumatic hip fractures admitted during the pandemic to pre-pandemic patients.

**Materials and methods:**

This retrospective cohort study at six level I trauma centers included patients with traumatic hip fractures. Patients with a non-fall mechanism and those who were transferred in were excluded. Patients admitted 16 March 2019–30 June 2019 were in the “pre-pandemic” group, patients were admitted 16 March 2020–30 June 2020 were in the “pandemic” group. The primary outcome was time from injury to arrival. Secondary outcomes were time from arrival to surgical intervention, hospital length of stay (HLOS), and mortality.

**Results:**

There were 703 patients, 352 (50.1%) pre-pandemic and 351 (49.9%) during the pandemic. Overall, 66.5% were female and the median age was 82 years old. Patients were similar in age, race, gender, and injury severity score. The median time from injury to hospital arrival was statistically shorter for pre-pandemic patients when compared to pandemic patients, 79.5 (56, 194.5) min vs. 91 (59, 420), *p* = 0.04. The time from arrival to surgical intervention (*p* = 0.64) was statistically similar between groups. For both groups, the median HLOS was 5 days, *p* = 0.45. In-hospital mortality was significantly higher during the pandemic, 1.1% vs 3.4%, *p* = 0.04.

**Conclusions:**

While time from injury to hospital arrival was statistically longer during the pandemic, the difference may not be clinically important. Time from arrival to surgical intervention remained similar, despite changes made to prevent COVID-19 transmission.

## Background

The USA issued national social distancing guidelines on 16 March 2020 in response to the coronavirus disease 2019 (COVID-19) pandemic [1, 2]. Social distancing has shown to be associated with decreased trauma admissions, whereas in some reports hip fractures admissions remained unchanged [3–7]. In one study, the number of proximal femur fragility fractures increased [7].

Concerns of contracting the highly contagious disease COVID-19 have led to reluctance in seeking medical care, which may be contributing to delayed hospital arrival for patients with traumatic injuries. The pandemic has caused psychological responses such as fear, especially in the geriatric population, which could be attributable to increased mortality for geriatric patients who contract COVID-19 [8–14]. Fear of COVID-19 may result in delayed arrival for patients with hip fractures, and this delay may disproportionately affect the geriatric population more than their younger cohorts [4]. Delayed time to hip fracture intervention has been shown to increase the risk for mortality [15–17]. The mortality rate among patients with hip fractures is already high and could be further affected by COVID-19 infection [18].

The study objective was to describe differences in the time from injury to hospital arrival for patients with traumatic hip fractures caused by a fall admitted during the pandemic to patients admitted pre-pandemic.

## Materials and methods

This multicenter retrospective cohort study was approved by the institutional review boards of the six participating level I trauma centers. Adult (age ≥ 18) patients with traumatic hip fractures were identified from the centers trauma registry using the International Classification of Diseases (ICD) version 10 codes beginning with ‘S72’. Patients with an injury mechanism other than a fall were excluded [23.0% of all hip fractures (*n* = 247)]. Only patients directly admitted to the participating hospitals’ emergency departments were included, patients transferred in from another treatment facility were excluded [14.5% of all hip fractures with an injury mechanism of a fall (*n* = 90)]. Patients were separated into two groups: admissions from 16 March 2019 to 30 June 2019 were in the “pre-pandemic” group and admissions from 16 March 2020 to 30 June 2020 were in the “pandemic” group.

All data was collected from the individual trauma center’s trauma registries. Variables required to be included in the trauma registry are outlined in the National Trauma Data Standard Data Dictionary created by the American College of Surgeons (ACS) who can provide verification of Level I trauma center status. In order to be designated Level I by the ACS, the hospitals must record the variables outlined in their data dictionary. However, individual centers and the state in which they reside may include more variables in their trauma registry at their discretion. Documentation of COVID-19 testing, and the patient’s testing results, is not required to be tracked by the ACS. However, of the six participating level I trauma centers, three trauma centers did track if the trauma patients were suspected of having COVID-19 or had a positive test result for COVID-19 in their trauma registry. The primary outcome was the time from injury to hospital arrival (minutes). Other outcomes included: time from arrival to surgical intervention (h), hospital length of stay (HLOS, days), and mortality.

A stratified analysis was conducted for geriatric patients (aged greater than or equal to 65 years old) and younger patients (aged less than 65 years old) because it was expected that any differences in time from injury to hospital arrival observed during the pandemic may be greater in the geriatric population. Another stratified analysis was conducted by in-hospital survival (alive and deceased) to examine associations between mortality and time from injury to hospital arrival.

Continuous variables were summarized as mean (standard deviation) or median (interquartile range) and were compared using Student’s *t* test or the Kruskal-Wallis test based on the distribution of data. Dichotomous and categorical variables were summarized using proportions (counts) and were compared using chi-squared or Fisher’s exact test, when appropriate. Logistic regression was used to identify predictors of mortality. Covariates which were different between pre-pandemic patients and pandemic patients at *p* < 0.20 were assessed for confounding. An alpha of 0.05 was used. In a post-hoc power analysis, the actual power for this study was 83%.

## Results

There was a total of 703 patients admitted, 352 (50.1%) pre-pandemic and 351 (49.9%) during the pandemic, the proportion of admissions due to a hip fracture was similar between groups, *p* = 0.14. Patients were similar in age, race, and injury severity score (ISS) (Table 1). There was a higher proportion of males admitted pre-pandemic compared to during the pandemic, 36.9% vs 30.2%, but this was not significant, *p* = 0.06. Admission labs and comorbidities were comparable between groups. The most common transportation mode was via ambulance for both groups, there were no statistical differences in the mode of transportation. There was also no statistical differences observed for the type of hip fracture [pertrochanteric (*p* = 0.58), femoral neck (*p* = 0.24), subtrochanteric (*p* = 0.13), or unspecified (*p* = 0.46)]. Additionally, the proportion of patients treated with surgical intervention was similar between groups, 88.4% pre-pandemic vs. 89.2% pandemic, *p* = 0.73. General reasons for non-operative management included the patient was deemed medically unstable and not cleared for surgery, the family or patient declined surgery and chose hospice instead, and there is a non-operative option available for patients with an impacted femoral neck.

**Table 1:** Patient demographics and clinical characteristics

	Pre-pandemic ***n*** = 352	Pandemic ***n*** = 351	***p*** value
**Age**^a^, median (IQR)	79.0 (71.0, 84.0)	78.0 (69.0, 85.0)	0.93
**Age above 89**, % (*n*)	18.6% (66)	19.4% (68)	0.83
**Aged less than 65**, % (*n*)	10.2% (36)	12.0% (42)	0.46
**Gender**, % (*n*) male	36.9% (130)	30.2% (106)	0.06
**Race**, % (n) White	90.6% (319)	87.2% (306)	0.15
**ISS**, median (IQR)	9 (9, 10)	9 (9, 10)	0.88
**ISS ≥ 16**, % (*n*)	4.3% (15)	4.0% (14)	0.86
**ISS < 16**, % (*n*)	95.7% (337)	96.0% (337)	
**Admission Labs**			
**SBP**, % (*n*) below 120 mmHg	13.6% (48)	18.0% (63)	0.12
**DBP**, % (*n*) below 80 mmHg	57.4% (202)	56.7% (199)	0.85
**HR**, % (*n*) below 100 bpm	88.9% (313)	89.5% (314)	0.82
**RR**, % (*n*) below 16	40.1% (138)	38.3% (132)	0.62
**SaO2**, % (*n*) below 95	57.7% (203)	62.4% (219)	0.20
**Temperature**, % (*n*) below 97 degrees F	85.5% (301)	86.6% (304)	0.67
**GCS**, % (*n*) equal to 15	79.3% (279)	80.9% (284)	0.58
**Comorbidity,** % (*n*) yes	88.9% (313)	91.7% (322)	0.21
**Comorbidity count**, median (IQR)	2 (1, 3)	2 (1, 3)	0.26
**Mode of transportation**^b^			
**Ambulance**, % (*n*)	98.7% (314)	99.7% (322)	0.21
**Private transportation**, % (*n*)	4.6% (16)	2.6% (9)	0.16
**Fixed wing**, % (*n*)	0.3% (1)	0.3% (1)	>0.99
**Helicopter**, % (*n*)	0.6% (2)	0.3% (1)	0.62
**Fracture type**			
**Pertrochanteric**, % (*n*)	40.6% (143)	42.2% (148)	0.58
**Femoral neck**, % (*n*)	48.0% (169)	43.6% (153)	0.24
**Subtrochanteric**, % (*n*)	1.4% (5)	3.1% (11)	0.13
**Unspecified femur fracture**, % (*n*)	9.9% (35)	11.7% (41)	0.46
**Surgical repair**, % yes (*n*)	88.4% (311)	89.2% (313)	0.73

*ISS* Injury Severity Score, *SBP* systolic blood pressure, *DBP* diastolic blood pressure, *mmHg* millimeters mercury, *bpm* beats per minute, *HR* heart rate, *RR* respiratory rate, *SaO2* oxygen saturation, *GCS* Glasgow coma scale

^a^Age greater than 89 years was aggregated into a single category

^b^Patients could have multiple modes of transportation

For both groups, the median HLOS was 5 days, *p* = 0.45 (Table 2). Complication rates for myocardial infarction (*p* > 0.99), stroke or cerebrovascular accident (*p* = 0.29), pulmonary embolism (*p* = 0.50), and deep vein thrombosis (*p* > 0.99) were all similar between groups. Discharge dispositions were statistically similar between groups; however, discharge destinations of skilled nursing facility and long-term acute care were trending toward significance (*p* = 0.06 and *p* = 0.06). There was a higher proportion of pre-pandemic patients than pandemic patients who were discharged to a skilled nursing facility, 24.7% vs 18.8%. There was also a higher proportion of pre-pandemic patients than pandemic patients discharged to long-term acute care, 10.5% vs 6.6%. There was a statistically significant difference for in-hospital mortality rates; 1.1% of pre-pandemic patients and 3.4% of pandemic patients died in-hospital, *p* = 0.04. Of the six centers participating in this study, only half (three) documented if the patient was tested for COVID-19 and the COVID-19 test results in their trauma registry; there were four confirmed and one suspected COVID-19 cases. None of the confirmed or suspected COVID-19 cases died in-hospital.

**Table 2:** Clinical outcomes

	Pre-pandemic ***n*** = 352	Pandemic ***n*** = 351	*p* value
**HLOS**, median (IQR)	5 (4, 6)	5 (3, 6)	0.45
**Total ventilator days**^a^, median (IQR)	2 (2, 9)	3 (2, 4)	0.86
**Complications**			
**Myocardial infarction**	0.6% (2)	0.6% (2)	> 0.99
**Stroke or CVA**	1.7% (6)	0.6% (2)	0.29
**Pulmonary embolism**	0.6% (2)	0% (0)	0.50
**Deep vein thrombosis**	1.1% (4)	0.9% (3)	> 0.99
**Discharge disposition**, % (*n*)			
**Home/home health**	6.5% (23)	9.4% (33)	0.16
**SNF**	24.7% (87)	18.8% (66)	0.06
**LTAC**	10.5% (37)	6.6% (23)	0.06
**Rehabilitation**	11.1% (39)	11.4% (40)	0.89
**Hospice**	2.3% (8)	4.6% (16)	0.10
**Other**	0.3% (1)	0.9% (3)	0.37
**In-hospital mortality**, % (*n*)	1.1% (4)	3.4% (12)	**0.04**

*HLOS* hospital length of stay, *SNF* skilled nursing facility, *Home/Home health* home or home with health care services

^a^Among patients ventilated

Among the patients who died in-hospital, the proportion of patients treated with surgical intervention was similar between pre-pandemic and pandemic patients, 75% (*n* = 3) vs. 67% (*n* = 8), respectively, *p* > 0.99. The time to death was not statistically different between pre-pandemic and pandemic patients, 4.5 days (0.1, 13.6) vs. 3.3 days (1.1, 6.5), *p* > 0.99. There was also no difference in the comorbidity count between groups among patients who died in-hospital, *p* = 0.14. Among those who died in-hospital, there was a lower proportion of pre-pandemic patients who were geriatric than among pandemic patients, 75% vs. 100%; however, this difference was not significant, *p* = 0.25. No pre-pandemic patients who died in-hospital experienced myocardial infarction, stroke, cerebrovascular accident, pulmonary embolism, or deep vein thrombosis. Whereas one pandemic patient who died in-hospital had a myocardial infarction and one other patient had a deep vein thrombosis.

**Fig. 1:**
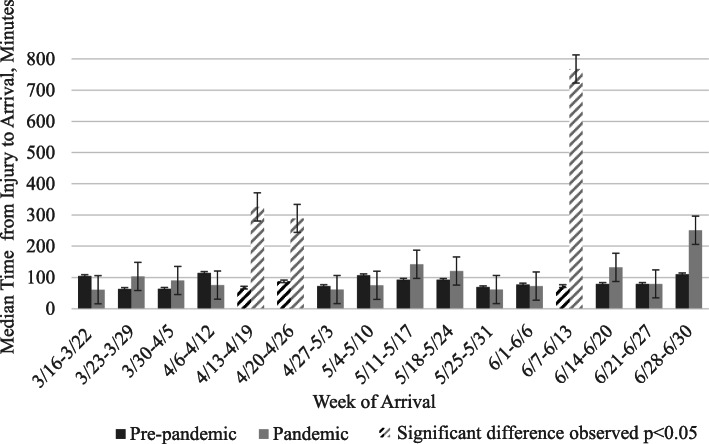
Median time from injury to arrival by week of arrival per study group

Figure 1 displays the median time from injury to hospital arrival by study admission week for each group. Pre-pandemic patients admitted experienced a shorter median time from injury to hospital arrival compared to the same corresponding week during the pandemic a majority of the time. However, there were only significant differences in the median time from injury to hospital arrival in mid-April (13 April–19 April and 20 April–26 April) and early June (7 June–13 June).

The overall median (full range) time from injury to hospital arrival was statistically shorter for pre-pandemic patients when compared to pandemic patients, 79.5 (31.4–848.0) vs. 91 (33.0–2930.0) min, *p* = 0.04 (Table 3). The median (full range) time from arrival to surgical intervention was similar between groups, 19.5 h (1.1–69.1) for pre-pandemic and 19.5 h (2.0–55.5) for pandemic patients, *p* = 0.64.

**Table 3:** Timing metrics for patients with hip fractures

	Pre-pandemic ***n*** = 352	Pandemic ***n*** = 351	***p*** value
**Time from injury to hospital arrival**, min, median (IQR)	79.5 (56.0, 194.5)	91.0 (59.0, 420.0)	**0.04**
Younger patients, age < 65 (*n* = 76)	96.0 (53.0, 251.0)	144.0 (67.0, 492.0)	0.18
Geriatric patients, age ≥ 65 (*n* = 613)	79.0 (57.0, 188.0)	86.0 (57.5, 402.0)	0.12
Survived (*n* = 673)	80.0 (57.0, 194.5)	92.0 (59.0, 449.0)	**0.05**
Died (*n* = 16)	42.0 (34.5 (212.5)	68.5 (54.0, 283.0)	0.14
**Time from arrival to surgical intervention**, h, median (IQR)	19.6 (12.3, 24.5)	19.5 (12.8, 23.3)	0.64
Younger patients, age < 65 (*n* = 76)	21.2 (12.4, 24.7)	14.3 (9.4, 19.6)	**0.01**
Geriatric patients, age ≥ 65 (*n* = 613)	19.5 (12.3, 24.3)	20.1 (13.7, 23.6)	0.54
Survived (*n* = 673)	19.6 (12.4, 24.4)	19.6 (13.1, 23.4)	0.69
Died (*n* = 16)	9.5 (2.0, 27.2)	13.8 (2.6, 26.3)	0.68

*COVID-19* coronavirus disease 2019

In a stratified analysis by age, there was no statistical difference in time from injury to hospital arrival by study period among younger patients, *p* = 0.18, or among geriatric patients, *p* = 0.12. The time from arrival to the surgical intervention was significantly longer among younger pre-pandemic patients compared to pandemic patients, 21.2 vs. 14.3 h, *p* = 0.01.

In a stratified analysis by mortality, patients who survived experienced a shorter time from injury to hospital arrival pre-pandemic than during the pandemic, *p* = 0.05. There were no differences between groups for time from injury to hospital arrival for patients who died in-hospital, *p* = 0.14. Time from arrival to surgical intervention was comparable between groups for both those who survived (*p* = 0.69) and those who died (*p* = 0.68). Across both pre-pandemic patients and pandemic patients, patients who survived experienced a longer time from arrival to surgical intervention than patients who died in-hospital.

The following variables were significant predictors of mortality: arrival during the pandemic, ISS, heart rate, Glasgow Coma Scale, and oxygen saturation (Table 4). Patients admitted during the pandemic were 3.1 (1.0, 9.6) times more likely to die in-hospital than patients admitted pre-pandemic. The time from injury to arrival was not a significant predictor of mortality. No variables were identified as confounding variables between the study groups (pandemic vs. pre-pandemic) and mortality.

**Table 4:** Predictors of mortality

Predictor variables	Mortality OR (CI)
Pandemic admission vs. pre-pandemic admission	**3.1 (1.0, 9.6)**
Time from injury to arrival	0.9 (0.8, 1.1)
Time from arrival to surgical intervention	0.9 (0.9, 1.0)
Female sex vs. male	0.4 (0.1, 1.0)
Age (> 65 years old vs. ≤ 65)	1.0 (0.2, 4.7)
ISS (> 9 vs. ≤ 9)	**3.9 (1.4, 10.9)**
Non-white race vs. white	0.5 (0.1, 4.1)
Comorbidity count	1.2 (0.9, 1.6)
Abnormal SBP vs. normal	0.8 (0.2, 2.9)
Abnormal DBP vs. normal	2.3 (0.8, 6.3)
Abnormal HR vs. normal	**5.3 (1.9, 15.0)**
Abnormal Temp vs. normal	1.4 (0.4, 5.1)
Abnormal GCS vs. normal	**2.4 (0.9, 6.9)**
Abnormal SaO2 vs. normal	**1.5 (0.6, 4.1)**

*OR* odds ratio, *CI* confidences interval, *COVID-19* coronavirus disease 2019, *ISS* injury severity score, *SBP* systolic blood pressure, *DBP* diastolic blood pressure, *HR* heart rate, *Temp* temperature, *GCS* Glasgow coma scale, *SaO2* oxygen saturation

## Discussion

This study demonstrated that while the time from hip fracture injury to hospital arrival was statistically longer during COVID-19, the difference was only 11.5 min and was not clinically profound. Substantial delays have been reported in other populations during COVID-19 due to fear; however, this may not be the case for patients with traumatic hip fractures who may not be mobile after their injury [13, 19–21]. Meanwhile the time from arrival to surgical intervention was similar for pre-pandemic patients and pandemic patients and was remarkably fast. We anticipated that the effect of a delayed time from injury to arrival would be greater among the geriatric population due to a potentially greater hesitation and reluctance to be an inpatient during the pandemic; however, there was no significant difference in the median time from injury to arrival among geriatric patients.

Few studies have reported the effect of the pandemic on the time from injury to arrival for patients with hip fractures [4, 22]. Dolci et al. examined the time from injury to emergency department access among all patients receiving an orthopedic and traumatology consultation, including patients with hip fractures, and reported that a lower proportion of patients had delayed arrival (more than 48 h) during lockdown (2%) when compared to 2019 (8%), *p* < 0.0001 [22]. In contrast to Dolci et al. in our study, there was a statistically longer time from injury to arrival among patients admitted during the pandemic; however, that difference was minor. Delayed hospital arrival due to fear of COVID-19 was reported by Minarro et al. among 19% of hip fracture patients causing an average delay of 2.5 days, considerably larger than the 11.5 min difference observed in this study [4]. When broken down by admission week, only certain admission weeks showed to have a significantly longer time from injury to arrival during the pandemic, in mid-April (13 April–26 April) and early June (7 June–13 June). Early in the pandemic after the lockdowns, it is possible patients were more scared of the virus and were more likely to stay in bed sick from the hip fracture rather than come into the hospital. As the first wave of the pandemic dissipated the difference in time from injury to hospital arrival between groups diminished. Another significant difference in the time from injury to arrival during the pandemic was observed in June and could be due to the increase in COVID-19 cases during this time, that may have again caused an increased fear of COVID-19 and hospital admission [23, 24]. While the overall difference in time from injury to hospital arrival was statistically significant, it may be not be clinically meaningful as time from injury to hospital arrival was not a significant predictor of mortality.

The average time from arrival to surgical intervention could also have been elongated during the pandemic, as protocols to protect the patient and provider from COVID-19 infection have been implemented and revised throughout the study period. Preoperative testing for COVID-19 was implemented at some centers; however, the testing procedures changed over time and were less common early in the pandemic when there were testing shortages. Patients known or suspected to have COVID-19 were treated in specific operating rooms, separate from patients without COVID-19. Ruggieri et al. also reported patients admitted to the emergency department and those requiring surgical treatment in an emergency were tested preoperatively but surgical treatment was not delayed for results [25]. Nevertheless, a statistically similar time from arrival to surgical intervention was observed between pre-pandemic and pandemic patients. In fact, younger pre-pandemic patients actually experienced a significantly longer time from arrival to surgical intervention than younger pandemic patients. This could be because elective procedures were canceled, so the participating trauma centers may have had more room to conduct surgical intervention for traumatic hip fractures more promptly during the pandemic. However, one study found that while elective procedures were canceled early in the pandemic, the number of elective hip fracture surgeries exceeded the number in 2019 by April 2020 [26]. The shorter time from arrival to surgical intervention for pandemic patients may only have been present among the younger patients, and not the geriatric patients, because the younger population may be stable and ready for surgical intervention sooner than the geriatric patients, who typically require more work up prior to surgery. Egol et al. reported also no difference in the time from arrival to surgical intervention for hip fracture patients during the pandemic [27]. Alternatively, Narang et al. reported a significant increase in the proportion of patients who experienced a delay to operation during the pandemic [28]. Two other studies also reported a significant increase in the time to surgery among patients admitted during the pandemic when compared to 2019 [29, 30]. Across both groups in this study, the median time from arrival to surgical intervention was consistent at 19.5 h, which is exceptionally faster that the current guideline recommendations. The American Academy of Orthopaedic Surgeons states there is moderate evidence to support the recommendation that achieving hip fracture surgery within 48 hours of admission is associated with better outcomes [31]. The trauma centers involved in this study aim to get patients to operating room within 24 h and were able to maintain that goal during the pandemic. Despite the lack of difference in time from arrival to surgical intervention between groups, the mortality rate during the pandemic was still triple that of the pre-pandemic period.

Several studies have reported an increased mortality rate among patients with hip fractures who were diagnosed with COVID-19 [3, 12, 18, 28, 32–36]. In this study, none of the confirmed or suspected COVID-19 patients died in-hospital so the COVID-19 diagnosis may not be driving this increased risk for mortality. Morelli et al. reported that COVID-19 may not be a contraindication for surgery in patients with proximal femur fragility fractures [37]. However, only three of the six centers reported the COVID-19 diagnosis in their trauma registries and the participating trauma centers did not have uniform testing criteria. Test criteria also changed throughout the study period among individual centers. Prior studies examining mortality of hip fractures during the pandemic report disparate findings; two other studies found a significant increase in mortality during the pandemic [27, 29], whereas three studies reported no difference in mortality among all patients [30, 38, 39]. We anticipated that an extended time from injury to arrival would increase mortality; however, time from injury to arrival was not identified as a statistically significant predictor of mortality regardless of the study time period. It is possible that if there were a longer significant delay, then the time from injury to hospital arrival could have been a significant predictor, but in this study the difference was only 11.5 min. Hadfield et al. discuss how patients treated in COVID-19-positive wards may be receiving less specialist input and reduced surgical specialty review; this change could contribute to increased mortality rates [3]. COVID-19-related stress and isolation during hospital course may also be contributing to lower resilience to recover. Interestingly, patients who died had a faster time from injury to arrival and faster time from arrival to surgical intervention than patients who lived within both populations (pre-pandemic and pandemic). This could indicate that those who were severely ill were not postponing arrival and that physicians were providing prompt evaluation and work up before surgery allowing for a fast time to surgical intervention. Despite that there was a significant increase in mortality, the overall count of in-hospital deaths (*n* = 16) was still relatively low. For both groups, the in-hospital mortality rate was still lower than other studies which examined in-hospital mortality prior to the pandemic [17, 40–43]. The COVID-19 mortality rate in this study was also lower than other similar studies which had mortality rates ranging from 4.8 to 12.3% during the pandemic [27, 29, 38, 39]. Especially because of the increased risk for mortality during the pandemic, it may be important for primary care providers should urge patients with known risk factors for falls, such as osteoporosis, to take extra precautions at home to help reduce the risk of hip fractures [5, 44].

### Limitations

This study had its limitations; it was a retrospective study over a short time period. COVID-19 diagnoses were not tracked in the trauma registries at all participating centers and there were few suspected/confirmed COVID-19 cases making investigation of COVID-19 as a predictor more difficult. The differences observed in delayed hospital arrival and greater mortality were associated with the pandemic period, but may not be causative or attributable to the pandemic. The time of injury may not have been exact and could have been estimated by the patient or emergency medical services. A physician at one trauma center involved in this study noted that COVID-19 was not causing a surge at their facility during the study time period; the results may have been different with a different time period. This study was conducted at level I trauma centers and may not be generalizable to other lower-level and non-trauma facilities. Additionally, the results may be different among areas which responded differently to the pandemic than the residing locations of the trauma centers in this study. The hospitals involved also responded to the pandemic individually and did not follow uniform procedures during the pandemic.

## Conclusions

While time from injury to hospital arrival was statistically longer during the pandemic, our hospitals reported a similar time from arrival to hip fracture surgical intervention. In fact, among younger patients, the time from arrival to hip fracture surgical intervention was faster during the pandemic. Remaining similar times to surgical repair was possible in spite of the pandemic and new and onerous protocols implemented to protect both the patient and the provider. However, mortality rates were significantly higher during the pandemic. While the difference in time from injury to hospital arrival was significantly longer, the difference of 11 min may not be clinically important and time from injury to hospital arrival was not a predictor of mortality.

## Data Availability

The datasets used and/or analyzed for the current study are available from the corresponding author on reasonable request.

## Abbreviations

COVID-19: Coronavirus disease 2019
ICD: International Classification of Diseases
HLOS: Hospital length of stay
ISS: Injury severity score
